# Intermittent screening and treatment with artemether–lumefantrine versus intermittent preventive treatment with sulfadoxine–pyrimethamine for malaria in pregnancy: a facility-based, open-label, non-inferiority trial in Nigeria

**DOI:** 10.1186/s12936-018-2394-2

**Published:** 2018-07-06

**Authors:** Ekpereonne Esu, Nicole Berens-Riha, Michael Pritsch, Nuria Nwachuku, Thomas Loescher, Martin Meremikwu

**Affiliations:** 1 Center for International Health (CIH), Ludwig-Maximilians-Universität (LMU), Leopoldstraße 7, 80802 Munich, Germany; 2 Department of Public Health, College of Medical Sciences, University of Calabar, Calabar, Nigeria; 3 Division of Infectious Diseases and Tropical Medicine, Medical Center of the University of Munich (LMU), Leopoldstrasse 5, 80802 Munich, Germany; 4 Department of Paediatrics, College of Medical Sciences, University of Calabar, Calabar, Nigeria

**Keywords:** Intermittent screening and treatment, Malaria in pregnancy, Intermittent preventive treatment, Artemether–lumefantrine, Sulfadoxine–pyrimethamine

## Abstract

**Background:**

The spread of SP resistance may compromise the effectiveness of intermittent preventive treatment of malaria in pregnancy (MiP) with sulfadoxine–pyrimethamine (IPTp-SP) across Africa. However, there is no recommended alternative medicine for IPTp or alternative strategy for prevention of MiP. This poses problems for the prevention of MiP. This study investigated, whether screening with a rapid diagnostic test for malaria at routine antenatal clinic attendances and treatment of only those who are positive (intermittent screening and treatment) with artemether–lumefantrine is as effective and safe as IPTp-SP in pregnant women.

**Methods:**

During antenatal clinic sessions at the General Hospital Calabar, Nigeria, held between October 2013 and November 2014, 459 pregnant women were randomized into either the current standard IPTp-SP or intermittent screening and treatment with artemether–lumefantrine (ISTp-AL). All women received a long-lasting insecticide-treated net at enrolment. Study women had a maximum of four scheduled visits following enrolment. Haemoglobin concentration and peripheral parasitaemia were assessed in the third trimester (36–40 weeks of gestation). Birth weight was documented at delivery or within a week for babies delivered at home.

**Results:**

In the third trimester, the overall prevalence of severe anaemia (Hb < 8 g/dl) and moderate (8–10.9 g/dl) anaemia was 0.8 and 27.7%, respectively, and was similar in both treatment groups (p = 0.204). The risk of third-trimester severe anaemia did not differ significantly between both treatment arms (risk difference − 1.75% [95% CI − 4.16 to 0.66]) although the sample was underpowered for this outcome due to several participants being unavailable to give a blood sample. The risk of third-trimester maternal parasitaemia was significantly lower in the ISTp-AL arm (RD − 3.96% [95% CI − 7.76 to − 0.16]). The risk of low birthweight was significantly lower in the ISTp-AL arm after controlling for maternal age, gravidity and baseline parasitaemia (risk difference − 1.53% [95% CI − 1.54 to − 1.15]). Women in the ISTp-AL arm complained of fever more frequently compared to women in the IPTp-SP arm (p = 0.022).

**Conclusions:**

The trial results suggest that in an area of high malaria transmission with moderate sulfadoxine–pyrimethamine resistance, ISTp with artemether–lumefantrine may be an effective strategy for controlling malaria in pregnancy.

*Trial registration* PACTR, PACTR201308000543272. Registered 29 April 2013, http://www.pactr.org/ATMWeb/appmanager/atm/atmregistry?dar=true&tNo=PACTR201308000543272

**Electronic supplementary material:**

The online version of this article (10.1186/s12936-018-2394-2) contains supplementary material, which is available to authorized users.

## Background

Malaria in pregnancy (MiP) remains a significant public health concern with over 32 million women at risk every year [1]. MiP has deleterious consequences for the mother and her fetus. Some of the well-known adverse effects include maternal anaemia, stillbirth, intrauterine growth retardation, low birth weight and preterm deliveries [2–4]. Malaria incidence and deaths have declined globally with about 214 million cases and 438,000 deaths reported in 2015 representing declines of 18 and 48% from the year 2000 [5]. These declines have been attributed to sustained scale-up and coverage of preventive interventions such as the use of long-lasting insecticide-treated nets (LLINs), vector control, prompt diagnosis with rapid diagnostic tests (RDTs) and effective treatment mainly with artemisinin-based combination therapy (ACT) [5, 6].

The World Health Organization (WHO) currently recommends a package of interventions to prevent MiP in areas with stable transmission in sub-Saharan Africa (SSA). These interventions include the use of LLINs, effective treatment and intermittent preventive treatment with sulfadoxine–pyrimethamine (IPTp-SP) given at scheduled antenatal visit beginning as early as possible in the second trimester [7].

Intermittent preventive treatment with sulfadoxine–pyrimethamine (SP) is very effective at reducing the adverse outcomes of MiP, but is threatened by the emergence of widespread parasite resistance [8, 9]. IPTp-SP has even been documented to cause harm by increasing placental proliferation of resistant parasites in pregnant women [10]. Mefloquine and a combination of azithromycin and chloroquine have been investigated as alternatives to SP for IPTp but were both poorly tolerated and thus not recommended for use in IPTp [11]. More recently, dihydroartemisinin-piperaquine (DHA-P) showed promising results as an alternative drug to replace SP for intermittent preventive treatment. However, further investigations are required to establish its safety and effects on birth outcome [12]. Thus there are currently no alternative drugs recommended for use in IPTp.

An alternative approach to IPTp-SP is intermittent screening and treatment in pregnancy (ISTp), which involves screening women for malaria with a rapid diagnostic test (RDT) during routine antenatal clinic visits and treatment of positives with an effective anti-malarial combination, e.g. an artemisinin-based drug combination. Two trials conducted in West Africa (Burkina Faso, Ghana, Mali, and The Gambia) have shown ISTp to be non-inferior to IPTp-SP in preventing low birth weight and maternal anaemia and placental malaria [11, 13]. Also, testing pregnant women for malaria before treatment encourages rational use of anti-malarial drugs and reduces drug pressure, which should slow down the development of parasite resistance.

This trial investigated whether ISTp-AL was non-inferior to IPTp-SP in the prevention of anaemia in pregnancy in an area of Nigeria with perennial malaria transmission.

## Methods

### Ethics

Participation in the study was voluntary; subjects were free to withdraw from the study at any time, and this did not affect their access to or quality of care. Information obtained from all subjects was treated as confidential. The trial was conducted under the provisions of the Declaration of Helsinki in its most recent form (2013) and in accordance with Good Clinical Practices guidelines set up by the WHO and by the International Conference on Harmonization.

The purpose of the study, the procedures to be followed and the potential risks, as well as benefits of participation, were explained to all participants. Information sheets and consent forms were provided to them for their review. Each participant signed an informed consent to participate in the research study.

The study protocol and informed consent forms were reviewed and approved by the Cross River Health Research Ethics Committee, Calabar, Nigeria and the Ethics Board of the Medical Center of the University of Munich (LMU), Munich, Germany. The trial was prospectively registered with the Pan African Clinical Trials Registry (PACTR201308000543272).

### Study design

This study was an individually randomized, two-arm, observer-blinded, parallel-group, non-inferiority trial. The trial was undertaken between October 2013 and November 2014 to investigate whether screening for malaria with RDT and treating all women with a positive result using AL was not inferior to intermittent preventive treatment with SP in the prevention of anaemia in pregnancy. Eligible and consenting pregnant women were randomly assigned (1:1) to one of the two study arms (1) LLIN plus IPTp-SP or (2) LLIN plus ISTp-AL.

### Study population

The study was conducted in Calabar, Cross River State in South-East Nigeria. In the 2006 Population and Housing Census, Cross River state was made up of 1,471,967 males and 1,421,021 females with an annual growth rate of 2.9%. The projected population for 2015 is 3,783,085.

The climate in Calabar is tropical-humid with wet and dry seasons, with average temperatures ranging between 15 and 30 °C and the annual rainfall between 1300 and 3000 mm. The vegetation in Calabar is mangrove swamp forest. Malaria transmission in this area is intense and perennial but with a peak in the rainy season, and *Plasmodium falciparum* is the predominant malaria-causing species [5, 14].

Previous studies have reported resistance to CQ and SP in Calabar to be over 80% [15]. *Anopheles gambiae* is the predominant vector species [16]. There is no information available on entomological inoculation rate (EIR) in the study area, but an EIR of about 259 infectious bites per person-year has been reported from Odukpani, a neighbouring area [17]. National HIV prevalence is reported to be about 3.4% with a higher prevalence of 5.5% in the region where the trial was conducted [18].

The trial was conducted at the antenatal clinics of the General Hospital in Calabar, Nigeria. The General Hospital is the largest government-owned secondary health facility in the city and caters to the health needs of the majority of the inhabitants. Since August 2009, pregnant women and children under 5 years of age receive free medical care as part of a funded welfare program by the Cross River State government. The average annual antenatal clinic attendance and births at the hospital are 16,550 and 3100 respectively. In the study area, the proportion of births attended by unskilled personnel has been estimated to be about 85% [19, 20]. In a health demographic and surveillance area 15 km from the trial site, only about 15% of births take place in health facilities [21].

The study population comprised pregnant women of all parities who presented at the antenatal clinics at their first booking. Antenatal care clinics were conducted 3 days per week; Wednesdays for first visits, then Mondays and Thursdays for follow-up visits. Women were screened for eligibility and invited to participate in the study if they met the criteria. Eligible participants were HIV-negative pregnant women between 16 and 24 weeks’ gestation at their first booking, no history of receiving intermittent preventive treatment with sulfadoxine–pyrimethamine during the pregnancy, resident in the study area, and willing to have a supervised delivery. Women with any illness requiring hospital admission (including severe malaria as defined by WHO), high-risk pregnancies, severe anaemia (Hb < 6 g/dl), known G6PD deficiency, a history of sensitivity to SP, lumefantrine or an artemisinin, or those unwilling to give consent were excluded.

### Sample size

The sample size was calculated based on the assumption that the prevalence of severe anaemia in the third trimester of pregnancy in the IPTp-SP arm of the study would be at least 3% based on findings from a previous study undertaken in West Africa [13]. Due to widespread resistance to SP, it was hypothesized that the prevalence of severe anaemia would be significantly lower in the LLIN plus ISTp-AL arm compared to the LLIN plus IPTp-SP arm. To establish that ISTp-AL was not inferior to IPTp-SP, it was necessary to show that the differences in the proportion of women with severe anaemia between both arms would not be more than 5%, a difference which would be of clinical and public health importance. To meet these criteria with 80% statistical power and allowing for 20% loss to follow-up it was calculated that 230 women were needed in each study arm giving a total sample size of 460.

### Procedures

All pregnant women presenting for their first antenatal care visit at the General Hospital were invited to attend group information sessions and were screened for their eligibility. After women had provided written informed consent, they were randomised to one of the two treatment groups (ISTp-AL or IPTp-SP arm) in computer-generated permuted blocks of ten. At enrolment, an eligible pregnant woman (16–24 weeks gestational age) was asked by a designated study nurse to pick a slip from the opaque envelope. The slip contained the treatment group the woman had been assigned to. The study arm women belonged to was not identifiable by the identification numbers given to them. Also, the principal investigator and outcome assessors (midwives and microscopist) were blinded to the randomization process and treatment allocation to prevent bias in outcome assessment. Women who declined to participate in the trial were treated with the routine standard of care, IPTp-SP according to the national guidelines. Enrolled study women were randomized to either the ISTp-AL or IPTp-SP arm.

All study women received a LLIN, which they were encouraged to use throughout the pregnancy. Also, they received a daily supplement of folic acid (4 mg) and ferrous sulphate (200 mg) tablets according to national guidelines. HIV screening was offered to all study women as part of the routine antenatal services recommended in Nigeria with an option for treatment, but the results of HIV screening were not available to the study team at the time of enrolment.

At enrolment, a finger prick blood sample was obtained for determination of haemoglobin concentration, preparation of blood smears (thin and thick films) for malaria parasite counts and preparation of dried blood spots (DBS) on filter paper. Women in the IPTp-SP arm received an initial dose of SP (1500 mg sulfadoxine/75 mg pyrimethamine) as a single dose. Pregnant women in ISTp-AL arm were screened for malaria infection with an SD Bioline rapid diagnostic test (RDT), a histidine-rich protein-2 (HRP-II) antigen and Plasmodium lactate dehydrogenase (pLDH) (Pan) antigen RDT kit. The RDTs were purchased from Codex Pharma Limited, Nigeria, as needed and stored following the manufacturer’s instructions. The tests were also performed and interpreted by the study team following the manufacturer’s instructions. Women in the ISTp-AL arm were treated with artemether–lumefantrine (20 mg artemether/120 mg lumefantrine) given as a 6-dose course, administered twice daily for 3 days if the RDT was positive. Study women were advised on the time and mode of administration for the 3 days treatment taken at home unobserved. Women in the ISTp-AL arm received no anti-malarial treatment if their RDT results were negative. Laridox^®^ (sulfadoxine–pyrimethamine, IPCA Laboratories Ltd, India) and Coartem^®^ (artemether–lumefantrine, Novartis Pharma, Switzerland) were used for this trial.

Study women were asked to return for follow-up antenatal care visits and IPTp-SP or screening with RDT at 24, 32 and 36 weeks of gestation if they were in the ISTp-AL group. At the next two follow-up visits (at 24 and 32 weeks of gestation), women in the IPTp-SP arm received SP while women in the ISTp-AL arm were screened with the RDT and, if positive, treated with AL. All women were followed-up by a study nurse through phone calls and at subsequent visits to document any complaints or adverse events.

Study women were also provided with a mobile phone number, which they could call to arrange unscheduled visits. Blood samples were obtained from all women in the third semester (36–40 weeks of gestation) before delivery for the determination of haemoglobin concentration and the preparation of blood smears. However, the smears were read retrospectively, and so the results were not available at the point of care. At delivery, placental smears were collected on microscopy slides to determine the prevalence of placental malaria. Study woman who came to the hospital with a history of fever or other symptoms of malaria between scheduled antenatal care visits was screened for malaria with RDT and if positive for malaria, treated with quinine (30 mg/kg daily for 5 days) regardless of treatment group according to national policy.

Birth weight was measured by a midwife who was unaware of the treatment group of the woman whom she was attending. The occurrence of miscarriages, stillbirths, neonatal deaths and the presence of congenital abnormalities were also recorded by midwives. Women were invited to re-attend at 6 weeks postpartum with their baby. A blood film was obtained from the mother. Her haemoglobin concentration was also measured at this time. Also, any neonatal adverse events were documented. A team of nurses, lab scientists was dedicated to the follow-up of women who did not present for delivery at the General hospital within 1 month of the estimated delivery date to establish pregnancy outcome.

### Laboratory evaluations

Labelled blood and impression smears were air-dried and stained with 4% Giemsa for 30 min. Thick smears were used to count the number of asexual parasites per 200 leukocytes, assuming 8000 leukocytes/μl of blood; slides were declared negative if no parasite was seen after examination of 100 high power fields. All blood films were read, and parasitaemia was quantified by two experienced and blinded microscopists independently. If there was a discrepancy in the findings in a slide between the two microscopists (positive or negative or a ≥ 50% difference in parasite density) a third, more senior microscopist read the slide and their reading was deemed to be the correct reading. Haemoglobin (Hb) concentration was measured using Hb 301 Hemocue (HemoCue AB, Angelholm, Sweden; accuracy of 0.1 g/dl).

### Adverse events monitoring and reporting

Case report forms were completed for maternal, and neonatal adverse events (AEs) detected at scheduled antenatal and postpartum visits, at unscheduled visits and delivery. The clinician on-call was alerted by the nursing team upon detection of a possible serious adverse event (SAE). AEs were considered SAEs if they fulfilled one of the following criteria: the event resulted in death, led to hospitalization, was a congenital abnormality, was life-threatening, caused disability, or was deemed serious for other medical reasons.

Serious adverse event reports were completed for study women who experienced miscarriages or stillbirths. Significant reductions in haemoglobin concentration (≤ 6 g/dl) after enrolment were also considered SAEs. Reports of drug side effects at each treatment course were considered as separate AEs.

### Statistical methods

Stata version 12 (StataCorp, College Station, Texas) was used for data analyses. The primary objective of the trial was to show that the risk of third-trimester severe anaemia (Hb ≤ 8 g/dl) in the ISTp-AL group was no more than 5% greater than in the IPTp-SP group. Secondary objectives were to demonstrate that the risks of low birth weight (< 2500 g), intrauterine deaths/stillbirths, spontaneous abortions, neonatal and maternal mortality were not significantly higher in women in the ISTp-AL group than in women who received IPTp-SP. The principal analysis of both primary and secondary outcomes was according-to-protocol (ATP), but a modified intention-to-treat (mITT) analysis was also undertaken. In the ATP analysis, only data from women who remained within their randomization group and had a record of the primary outcome were included. This implied that study women had to have received two courses of SP (IPTp-SP arm) or been screened twice using an RDT at scheduled visits (ISTp-AL arm) and also, had a measurement for the primary outcome (haemoglobin level at 36–40 weeks gestation).

In the modified intent-to-treat analysis (mITT), data from women who received an initial treatment of IPTp or had an initial screening test done and had a record for the primary outcome or the outcome of interest recorded was included. This implied that study women had to have received one course of SP (IPTp-SP arm) or been screened once using an RDT at scheduled visits (ISTp-AL arm) and also, have a measurement for the primary outcome or outcome of interest.

The proportion of ATP and mITT populations experiencing each outcome for the trial arms, and the associated 2-sided 95% CI for the risk difference, was estimated using the generalized linear model. To declare non-inferiority with a significance level of 0.05, the upper boundary of the 2-sided 95% CI for the estimated treatment effect had to be below the pre-defined non-inferiority margin (∆) of 5%. Gestational age at enrolment, being primigravidae, baseline anaemia and parasitaemia were controlled for using binomial regression.

Only birth weights from singleton pregnancies of live births ≥ 22 weeks’ gestation with no congenital abnormality and measured within 6 days of delivery were included in birth weight analyses. p-values less than 0.05 were considered statistically significant.

## Results

### Baseline demographic and clinical characteristics

**Fig. 1:**
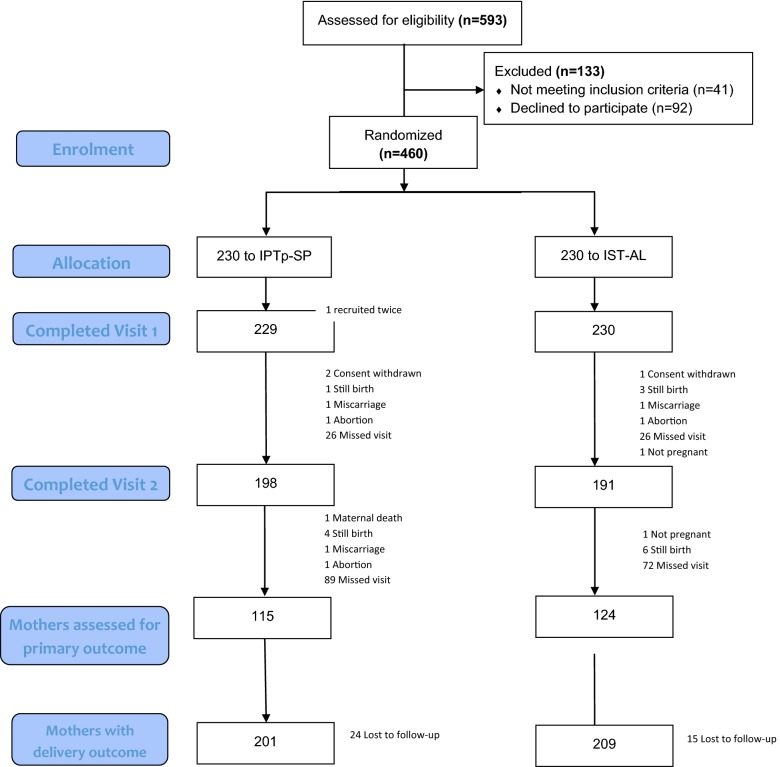
Trial profile showing enrolment and follow-up status of study women

A total of 460 of the 593 (77.6%) potentially eligible pregnant women who were screened were enrolled into either the IPTp-SP arm or the ISTp-AL arm (Fig. 1). Of the 133 women excluded at the screening stage, 41 did not meet inclusion criteria while 92 women declined participation in the study. 459 study women completed the first visit (i.e. received an initial treatment of IPTp with SP or had had an initial screening test done).

Baseline characteristics of study women in both treatment groups were very similar at enrolment (Table 1). About three-quarters of the study women in both arms were aged between 13 and 30 years with an overall mean age of 28.2 years. All study women had some level of formal education, and over 90% of them had been educated up to at least secondary level.

**Table 1:** Comparison of demographic and baseline characteristics of study women

Variable	SP-IPTp		ISTp-AL		Total	
	(N = 229)		(N = 230)		(N = 459)	
	n	(%)	n	(%)	n	(%)
Age (years)						
≤ 25	58	25.3	76	33	135	29.4
26–30	103	45	86	37.4	189	41.0
≥ 31	68	29.7	68	29.6	136	29.6
Mean (SD)	28.4	(4.6)	27.9	(5.4)	28.2	(5.0)
Median (IQR)	28	(6)	28	(7)	28	(6)
Educational attainment						
Primary	7	3.1	6	2.6	13	2.8
Secondary	96	41.9	99	43	195	42.5
Tertiary	126	55	125	54.4	251	54.7
Occupation						
None	10	4.4	21	9.1	31	6.8
Student	49	21.4	46	20	95	20.6
Housewife	26	11.4	24	10.4	50	10.9
Small business owner	92	40.1	90	39.1	182	39.7
Salary worker	52	22.7	49	21.3	101	22
Gravidity						
Primagravidae	107	46.7	109	47.4	216	47.0
Secundigravidae	68	29.7	69	30.0	137	29.9
Multigravidae	54	23.6	52	22.6	106	23.1
Household ownership of bed net						
No	99	43.2	108	47	207	45.1
Yes	130	56.8	122	53	252	54.9
Slept under bed net (previous night)						
No	197	86.	195	84.8	392	85.4
Yes	32	14	35	15.2	67	14.6
Baseline parasitaemia						
No	212	92.6	206	89.6	418	91.1
Yes	17	7.4	15	6.5	32	7.0
Parasite density geometric mean [95% CI]	954.8	[490.4–1859.3]	951	[452.5–1998.5]	953	[597.3–1520.6]
Haemoglobin (g/dl)						
≥ 11	135	59	165	71.7	300	65.4
8–10.9	92	40.2	62	27.0	154	33.5
< 8	2	0.8	3	1.3	5	1.1
Mean (SD)	11.4	(1.4)	11.6	(1.3)	11.5	(1.4)
Median (IQR)	11.3	(1.8)	11.6	(1.4)	11.5	(1.6)

The majority of the study women were students in tertiary institutions. Only 22% of the study women were salary workers comprised mainly of civil servants and teachers. Fifty-seven percent of the study population households already owned a bed net. However self-reported bed net use the night preceding enrolment was about 15%. Forty-seven percent of study women were primigravidae, and 29% were multigravidae.

1.1% of women had a haemoglobin concentration below 8 g/dl; the overall mean Hb concentration was 11.5 g/dl. Asymptomatic parasitaemia as determined by microscopy was found in 7.0% (32/459) of study women overall with parasite densities less than 500/ml in about 37.5% (12/32). The PCR-adjusted malaria prevalence at enrolment was 6.3% (29/459). The prevalence of malaria infection at enrolment in women in the ISTp-AL arm as determined by the SD Bioline^®^ rapid diagnostic test was 11.3% (RDTs were not done in women in the IPTp-SP arm). Baseline anaemia was significantly associated with asymptomatic malaria parasitaemia (p < 0.001). *Plasmodium falciparum* was the common malaria species. One mixed infection of *Plasmodium malariae* and *P. falciparum* was observed. The overall prevalence of HIV in pregnant women who were enrolled during the period of the trial was 1.3%

### Study outcomes-according to protocol (ATP) analysis

At the end of follow-up, 239 (51.9%) evaluable records for third-trimester haemoglobin concentration and 329 (71.5%) records for birth weight were available. Information on the outcome of pregnancy was obtained for 420 of the 459 study women (91.5%), 331 (72.1%) of whom delivered at a health centre or hospital and 58 (12.6%) at home. Three women withdrew from the study (two in the IPTp-SP arm and one in the ISTp-AL arm).

In the third trimester before delivery, the overall prevalence of severe anaemia (Hb < 8 g/dl) and moderate (8–10.9 g/dl) anaemia was 0.8 and 27.7% respectively (Table 2) and was similar in both treatment groups (p = 0.142). Throughout pregnancy, the overall prevalence of asymptomatic parasitaemia based on microscopy and PCR were 4 and 4.2%, respectively.

**Table 2:** Comparison of the main outcomes in women enrolled in IPTp-SP and ISTp-AL arms (at 36–40 weeks gestation and birth)

Outcome	ATP analysis			mITT analysis		
	Control (IPTp-SP)	Intervention (ISTp-AL)	p-value	Control (IPTp-SP)	Intervention (ISTp-AL)	p-value
Severe anaemia (Hb < 8 g/dl)	2/114 (1.7)	0/124 (0)	*0.133*	2/115 (1.7)	0/124 (0)	*0.142*
Moderate anaemia (Hb 8–10.9 g/dl)	36/114 (31.6)	30/124 (24.2)		36/115 (31.3)	30/124 (24.2)	
Hb ≥ 11 g/dl	76/114 (66.7)	94/124 (75.8)		77/115 (67)	94/124 (75.8)	
Mean haemoglobin g/dl (SD)	11.4 (1.3)	11.7 (1.2)	*0.073*	11.4 (1.4)	11.7 (1.2)	*0.076*
Birth weight						
Normal (≥ 2.5 kg)	130/139 (93.5)	139/147 (94.6)	*0.712*	148/158 (93.7)	157/167 (94)	*0.898*
Low (< 2.5 kg)	9/139 (6.5)	8/147 (5.4)		10/158 (6.3)	10/167 (6.0)	
Mean (SD)	3.21 (0.51)	3.18 (0.54)	*0.062*	3.21 (0.53)	3.17 (0.53)	*0.497*
Median (IQR)	3.2 (0.6)	3.2 (0.6)		3.2 (0.6)	3.2 (0.6)	
Parasitaemia						
Peripheral blood (light microscopy) All infections	4/96 (4.2)	2/112 (1.8)	*0.304*	4/96 (4.2)	2/112 (1.8)	*0.304*

**Fig. 2:**
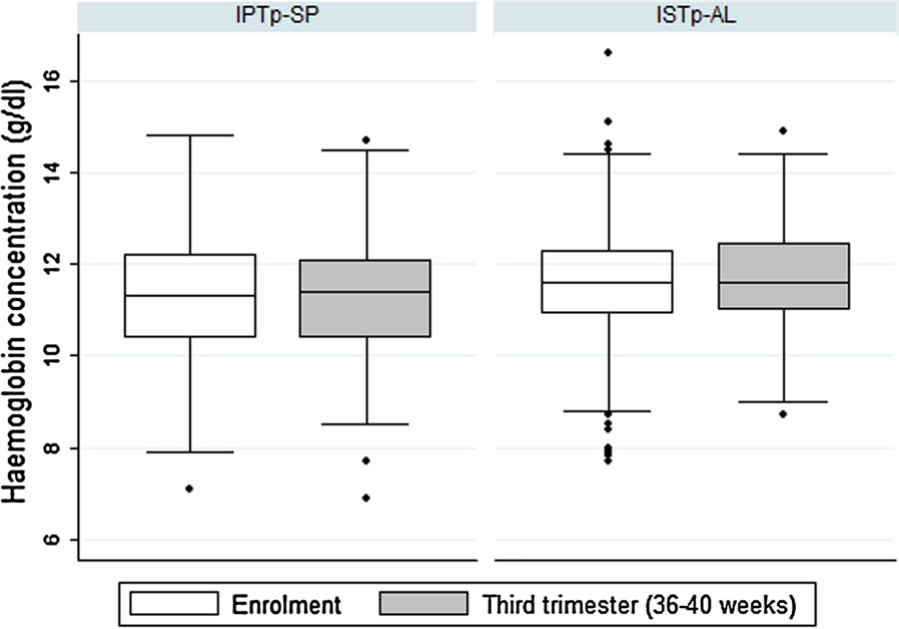
Variation of haemoglobin concentration between enrolment and third trimester (36–40 weeks gestation)

The mean haemoglobin concentration of all women between 36 and 40 weeks of gestation was 11.5 g/dl. The mean HB concentration in the ISTp-AL and IPTp-SP groups were 11.7 and 11.4 g/dl, respectively. However, this difference was not statistically significant (p = 0.073). There was a non-significant increase in mean haemoglobin concentration of 0.05 g/dl at 36–40 weeks of gestation over the baseline haemoglobin concentration (p = 0.54) (Fig. 2). There was no significant difference in haemoglobin concentration during the course of pregnancy between the two trial arms. The risk of third-trimester severe anaemia (Hb < 8 g/dl) did not differ significantly between both treatment groups (risk difference − 1.75% [95% CI − 4.16 to 0.66]. The upper boundary of the 2-sided 95% CI for the risk difference estimated between the ISTp-AL and IPTp-SP groups was below the non-inferiority margin of 5% set for third-trimester severe anaemia.

The risk of parasitaemia in the third trimester (36–40 weeks of gestation) was significantly higher in the IPTp-SP arm than in the ISTp-AL arm (Table 2). The risk difference for parasitaemia between the ISTp-AL and IPTp-SP groups was − 3.96% [95% CI − 7.76 to − 0.16]. One episode of illness associated with malaria parasitaemia was recorded in the IPTp-SP arm, throughout the trial. In all 531 RDT tests that were done in women in the ISTp-AL treatment group, a total of 37 (7%) were positive and led to treatment.

The prevalence of placental malaria at delivery as determined by microscopy was low with only one low-density infection found out of 46 (10%) samples collected from study women.

The overall prevalence of low birth weight was 5.9% in the study women and did not differ significantly between the two trial groups, although the trial was not powered to investigate non-inferiority for this outcome (Table 2). The risk difference for low birthweight between the ISTp-AL and IPTp-SP arms was (risk difference − 1.03% [95% CI − 6.53 to 4.46]. In an adjusted analysis which controlled for maternal age, gravidity and baseline parasitaemia, the risk difference for low birthweight was (risk difference − 1.53% [95% CI − 1.54 to − 1.15] and significantly lower in the ISTp-AL arm (p < 0.001).

The risk of anaemia (severe or moderate) was comparable between both treatment groups (Table 2). Primigravidae women were significantly associated with a lower risk of anaemia compared to secundi- and multigravidae in univariate analysis. In a multivariate analysis, which adjusted for baseline anaemia, treatment group, maternal age, and baseline parasitaemia, primigravidae remained significantly associated with lower risk of anaemia in the third trimester (Table 3).

**Table 3:** Factors associated with third-trimester anaemia (< 11 g/dl) in study women (ATP analysis)

	Unadjusted Risk Ratio	(95% CI)^a^	p-value^b^	Adjusted Risk Ratio	(95% CI)	p-value
Treatment group						
ISTp-AL	0.83	0.57–1.20	*0.318*	0.81	0.57–1.17	*0.259*
IPTp-SP	1			1		
Age category						
≤ 25	1			1		
26–30	1.07	0.67–1.72	*0.766*	0.95	0.59–1.52	*0.824*
≥ 31	1.31	0.81–2.12	*0.279*	1.03	0.61–1.75	*0.899*
Baseline parasitaemia						
Yes	1.49	0.90–2.45	*0.119*	1.46	0.89–2.41	*0.138*
No	1			1		
Gravidity						
Primigravidae	0.57	0.38–0.87	*0.008*	0.59	0.37–0.93	*0.024*
Secundigravidae	0.67	0.42–1.08	*0.104*	0.66	0.41–1.06	*0.087*
Multigravidae	1			1		

*CI* confidence interval

^a^p ≤ 0.05 means observed differences between comparison groups is statistically significant or not significant if p > 0.05

^b^Risk ratios were modelled using binomial regression. Treatment group, age category, baseline parasitaemia and gravidity were included in the final model

The risk of moderate anaemia (< 11 g/dl) was similar in the ISTp-AL group compared to IPTp-SP; (RD = − 6.1% [95% CI − 17.9 to 5.8]. In the adjusted analysis, the risk difference was − 5.4% [95% CI − 17.0 to 6.2]. All malaria infections were mild, and thus no study woman was admitted to hospital.

The risk of low birth weight did not differ significantly between both trial arms regardless of gravidity (Table 2). Baseline parasitaemia in pregnant women was not significantly associated with the low birth weight of their babies (Table 4). None of the study women with low birthweight babies had severe anaemia at enrollment.

**Table 4:** Factors associated with low birth weight of babies delivered by study women (ATP analysis)

	Unadjusted RR	(95% CI)	p-value^a^	Adjusted RR^b^	(95% CI)	p-value
Treatment group						
ISTp-AL	0.84	0.33–2.12	*0.712*	0.84	0.33–2.17	*0.72*
IPTp-SP	1			1		
Age category						
≤ 25	1			1		
26–30	1.17	0.37–3.77	*0.787*	1.43	0.44–4.59	*0.548*
≥ 31	1.08	0.30–3.86	*0.911*	2.22	0.58–8.42	*0.242*
Baseline parasitaemia						
Yes	1.46	0.35–5.99	*0.603*	1.62	0.39–6.63	*0.505*
No	1					
Baseline mild anaemia						
Yes	1.32	0.52–3.37	*0.558*	1.41	0.53–3.70	*0.489*
No	1					
Gravidity						
Primigravidae	3.18	0.73–13.81	*0.123*	4.54	0.94–21.84	*0.059*
Secundigravidae	1.31	0.23–7.65	*0.761*	1.44	0.24–8.69	*0.694*
Multigravidae	1					

*CI* confidence interval

^a^p ≤ 0.05 means observed differences between comparison groups is statistically significant or not significant if p > 0.05

^b^Risk ratios were modelled using binomial regression. Treatment group, age category, baseline parasitaemia and mild anaemia, and gravidity were included in the final model

The risks of third-trimester anaemia and low birth weight in women who were RDT negative throughout pregnancy and did not receive AL treatment were similar to that of women in the IPTp-SP arm (data not shown, see Additional file 1). The most frequent complaints since the last ANC visit by study women were headache, cough, catarrh, vomiting and dizziness. These symptoms were not significantly different between both treatment groups. However, women in the ISTp-AL arm complained of fever since their last antenatal clinic visit more frequently than in the IPTp-SP arm (p = 0.022) (Table 5). Of the nine participants that had a fever, seven were in the ISTp-AL arm. Three of these seven participants in the ISTp-AL arm had malaria infections at the time.

**Table 5:** Comparison of number of women who experienced adverse events

	IPTp-SP	ISTp-AL	p-value	Total
	n (%)	n (%)		n (%)
Headache	35 (15.3)	34 (14.8)	*0.929*	69 (15)
Cough	23 (10)	17 (7.4)	*0.323*	40 (8.7)
Catarrh	15 (6.6)	15 (6.5)	*0.966*	30 (6.5)
Vomiting	7 (3.1)	9 (3.9)	*0.641*	16 (3.5)
Dizziness	6 (2.6)	10 (4.3)	*0.319*	16 (3.5)
Fever	2 (0.9)	10 (4.3)	*0.022*	12 (2.6)
Body pain	5 (2.2)	4 (1.7)	*0.699*	9 (2.0)
Itching	4 (1.7)	1 (0.4)	*0.172*	5 (1.1)
Nausea	1 (0.4)	–	*0.337*	1 (0.2)
Rash	–	1 (0.4)	*0.338*	1 (0.2)
Sleepiness	–	1 (0.4)	*0.338*	1 (0.2)

There were no statistically significant differences for preterm deliveries, abortions or perinatal deaths between both study arms (Additional file 2). There was one maternal death in the SP-IPTp arm. The death occurred after her second visit at 30 weeks gestation. It was not possible to establish the cause of death. No drug-related serious adverse event occurred.

### Modified intention to treat analysis

All the primary and secondary outcomes were also analysed using modified intention to treat analysis with similar results to those of the ATP analysis. For the key outcomes of anaemia and low birth weight, there were no significant differences as with ATP analysis (Table 2). The overall prevalence of parasitaemia at 36–40 weeks gestation was 1.88%. There was also significantly higher parasitaemia in the IPTp-SP arm compared to the ISTp-AL arm (p = 0.034). Factors associated with third-trimester anaemia and low birth weight are included in additional data (Additional files 3 and 4).

## Discussion

This trial has demonstrated that ISTp using AL was not inferior to IPTp with SP in preventing severe maternal anaemia. The study also showed a significantly lower risk of parasitaemia in pregnant women and low birth weight in newborns of mothers in the ISTp-AL arm. The incidence of preterm deliveries, abortions and perinatal deaths was similar in both study groups.

About 43% of study women did not contribute data on third-trimester anaemia (primary outcome) but contributed data to the birth weight analysis. This is attributable to several national strikes embarked upon by different cadres of health workers. These adversely affected follow-up visits and the recording of related outcomes as the health facility was locked up over these periods. Thus, the results obtained for third-trimester anaemia are likely not powered to exclude clinically significant differences based on the sample size calculation, and non-inferiority margin set a priori. However, the ATP and mITT analyses yielded similar results and loss to follow-up was 8.5%, and there was no significant difference in numbers of women lost to follow-up between the treatment arms.

The overall incidence of malaria and severe anaemia in study women was observed to be much lower than expected and relative to previously reported estimates [22–24]. All the malaria infections among study women were mild with none requiring hospital admission. Apart from the fact that pregnant women often have low-density asymptomatic malaria infections this finding may be explained by continued decline in malaria cases and the sustained scale-up in coverage of preventive interventions, including LLIN. All study women received LLIN at enrolment and were encouraged to continue sleeping under them at scheduled follow-up visits. The Cross River State government has also provided free medical care to pregnant women and under-fives since 2009.

The ISTp intervention was well received by most pregnant women and health providers in this study. This is similar to findings from a trial in West Africa [25]. However, a few study women withdrew consent from this study as a result of the repeated blood draws and finger pricks. However, ISTp-AL is likely to be more expensive than IPTp-SP because of the cost of RDTs and doses of artemether–lumefantrine, which are higher than the cost of a dose of SP. AL is also more complex to administer being a three-day treatment. Thus, adherence monitoring is more difficult and would mostly rely on self-report by pregnant women or home visits by health workers.

The transmission of malaria parasites from humans to mosquitoes requires the presence of infectious gametocytes in the human peripheral blood. Consequently, pregnant women with asymptomatic parasitaemia could constitute a reservoir of parasites for the inoculation of mosquitoes [26]. It is important to note that women in the IPTp-SP treatment group had a higher risk of parasitaemia compared to women in the ISTp-AL treatment group. AL has been shown to be more effective at reducing gametocyte carriage and malaria transmission to mosquitoes compared to DHA-P [27]. ISTp with AL or other ACT regimens could reduce the incidence of malaria and anaemia, which could bring immense health and socioeconomic benefits. These advantages could be considered to outweigh the lower cost-effectiveness of the approach in the long term.

Previous trials [11, 13, 28, 29] have investigated alternative drug regimens and strategies to replace SP for intermittent preventive treatment of malaria in pregnancy. The most promising results are from Desai et al. [12] who found that IPTp with DHA-P was more effective than ISTp with DHA-P or IPTp with SP at preventing peripheral or placental malaria at delivery. This result suggests that ISTp as a strategy may not be suitable for settings with high transmission. A recently published trial [30], that evaluated four artemisinin-based combinations for the treatment of African pregnant women (second and third trimester) with malaria and found DHA-P to have the best efficacy and an acceptable safety profile. However, AL was associated with the fewest adverse effects and an acceptable cure rate.

Moreover, the current levels of RDT sensitivity suggest that the performance of presently available tests is inadequate for the screening of asymptomatic women who typically have low-density infections that are often missed by RDTs [12, 31]. There is also a concern about the absence of a prophylactic effect in women who test negative. This allows low-density infections to persist and increases the chance of new infections occurring between scheduled antenatal visits.

The WHO recently recommended the continued use of IPTp-SP for the prevention of MiP as there is not sufficient evidence to suggest that it be replaced by ISTp or any other drug regimen replacing SP for use as IPTp [32].

ISTp remains a valuable approach to the control of MiP that needs to be further explored. IST may have other malaria control applications in endemic countries as they approach pre-elimination phase of malaria control, characterized by low-density infections in asymptomatic carriers. The intermittent screening may serve as a malaria surveillance tool for monitoring population malaria levels. ISTp as an approach to control malaria in pregnancy may be more effective when highly sensitive RDTs become available and if treatment can be with a single dose regimen, preferably produced from non-artemisinin derivatives, but effective against gametocyte. This will ensure therapy can be directly observed at the antenatal clinic. The use of a non-artemisinin-based drug would prevent the potential development of artemisinin resistance.

## Conclusions

This study showed non-inferiority of ISTp-AL to IPTp-SP for preventing maternal anaemia in pregnant women in an area of high malaria transmission and moderate SP resistance. ISTp remains one of the potential alternatives to IPTp-SP, and its relevance will become more evident when the sensitivity of current generation of RDTs are improved beyond present levels. In the meantime, high priority must be given to identifying safe and cost-effective alternatives to SP for use in IPTp.

## Additional files

**Additional file 1.** Anaemia before delivery (36-40 weeks) and birth weight by treatment group in women who were RDT negative throughout the trial and received no anti-malarial drug.

**Additional file 2.** Comparison of delivery outcomes for singleton births.

**Additional file 3.** Factors associated with third-trimester anaemia (<11g/dl) in study women (mITT analyses).

**Additional file 4.** Factors associated with low birth weight of babies delivered by study women (mITT analyses).

## Abbreviations

ATP: according to protocol
AE: adverse event
ANC: antenatal care
AL: artemether–lumefantrine
ACT: artemisinin-based combination therapy
G6PD: glucose-6-phosphate dehydrogenase
Hb: haemoglobin
HRP-II: histidine-rich protein II
HIV: human immunodeficiency virus
ITN: insecticide-treated net
IPT: intermittent preventive treatment
IST: intermittent screening and treatment
LLIN: long-lasting insecticide-treated net
MiP: malaria in pregnancy
mITT: modified intention to treat analysis
pLDH: Plasmodium lactate dehydrogenase
RDT: rapid diagnostic test
SAE: serious adverse event
SP: sulfadoxine–pyrimethamine
WHO: World Health Organization
