# Supplementary material for: Intermittent screening and treatment with artemether–lumefantrine versus intermittent preventive treatment with sulfadoxine–pyrimethamine for malaria in pregnancy: a facility-based, open-label, non-inferiority trial in Nigeria
Source: Malar J. 2018 Jul 6;17:251. doi: 10.1186/s12936-018-2394-2 (PMC6034215; doi:10.1186/s12936-018-2394-2)
Supplement: Supplementary file 1 — Additional file 1. Anaemia before delivery (36-40 weeks) and birth weight by treatment group in women who were RDT negative throughout the trial and received no anti-malarial drug. [file 12936_2018_2394_MOESM1_ESM.docx]

**Additional file 1: Anaemia before delivery (36-40 weeks) and birth weight by treatment group in women who were RDT negative throughout the trial and received no anti-malarial drug**

| **Outcome** | **Per protocol analysis** | | |
| --- | --- | --- | --- |
| **Anaemia** | **Control**  **(IPTp-SP)** | **Intervention**  **(ISTp-AL)** | **p-value** |
| Severe anaemia  (Hb< 8g/dl) | 2/114 (1.7) | 0/84 (0) | **0.23** |
| Moderate anaemia  (Hb 8-10.9 g/dl) | 36/114 (31.6) | 20/84 (23.8) |  |
| Hb ≥ 11 g/dl | 76/114 (66.7) | 64/84 (76.2) |  |
| Mean haemoglobin g/dl (SD) | 11.4 (1.3) | 11.7 (1.17) | **0.096** |
| **Birth weight** |  |  |  |
| Normal (≥ 2.5kg) | 130/139 (93.5) | 62/65 (95.4) |  |
| Low (< 2.5kg) | 9/139 (6.5) | 3/65 (4.6) |  |
| Mean (SD) | 3.21 (0.51) | 3.23 (0.43) | **0.753** |
| Median (IQR) | 3.2 (0.6) | 3.2 (0.5) |  |
