# Supplementary material for: Intermittent screening and treatment with artemether–lumefantrine versus intermittent preventive treatment with sulfadoxine–pyrimethamine for malaria in pregnancy: a facility-based, open-label, non-inferiority trial in Nigeria
Source: Malar J. 2018 Jul 6;17:251. doi: 10.1186/s12936-018-2394-2 (PMC6034215; doi:10.1186/s12936-018-2394-2)
Supplement: Supplementary file 2 — Additional file 2. Comparison of delivery outcomes for singleton births. [file 12936_2018_2394_MOESM2_ESM.docx]

**Additional file 2: Comparison of delivery outcomes for singleton births**

|  | IPTp-SP  n (%) | ISTp-AL  n (%) | p-value | Total  n (%) |
| --- | --- | --- | --- | --- |
| Term deliveries | 191 (93.2) | 198 (92.1) |  | 389 (92.6) |
| Preterm deliveries | 1 (0.48) | - | 0.3056 | 1 (0.24) |
| Abortions | 2 (0.96) | 2 (0.93) | 0.9618 | 4 (0.95) |
| Perinatal deaths | 11 (5.36) | 15 (6.97) | 0.4932 | 26 (6.2) |
