# Supplementary material for: Intermittent screening and treatment with artemether–lumefantrine versus intermittent preventive treatment with sulfadoxine–pyrimethamine for malaria in pregnancy: a facility-based, open-label, non-inferiority trial in Nigeria
Source: Malar J. 2018 Jul 6;17:251. doi: 10.1186/s12936-018-2394-2 (PMC6034215; doi:10.1186/s12936-018-2394-2)
Supplement: Supplementary file 3 — Additional file 3. Factors associated with third-trimester anaemia (<11g/dl) in study women (mITT analyses). [file 12936_2018_2394_MOESM3_ESM.docx]

**Additional file 3: Factors associated with third-trimester anaemia (<11g/dl) in study women (mITT analyses)**

|  | **Unadjusted Risk Ratio** | **(95%CI)^a^** | **p-value^b^** | **Adjusted Risk Ratio** | **(95%CI)** | **p-value** |
| --- | --- | --- | --- | --- | --- | --- |
|  |  |  |  |  |  |  |
| **Treatment group**  **ISTp-AL**  **IPTp-SP** | 0.83  1 | 0.58 to 1.21 | **0.341** | 0.82  1 | 0.57 to 1.17 | **0.272** |
| **Age category**  **≤ 25**  **26-30**  **≥ 31** | 1  1.06  1.31 | 0.66 to 1.71  0.81 to 2.12 | **0.798**  **0.279** | 1  0.94  1.03 | 0.58 to 1.05  0.61 to 1.75 | **0.785**  **0.904** |
| **Baseline parasitaemia**  **Yes**  **No** | 1.49  1 | 0.91 to 2.46 | **0.115** | 1.45  1 | 0.89 to 2.43 | **0.128** |
| **Gravidity**  **Primigravidae**  **Secundigravidae**  **Multigravidae** | 0.57  0.66  1 | 0.38 to 0.87  0.41 to 1.07 | **0.008**  **0.092** | 0.59  0.65  1 | 0.37 to 0.93  0.40 to 1.05 | **0.024**  **0.076** |
