# Supplementary material for: Intermittent screening and treatment with artemether–lumefantrine versus intermittent preventive treatment with sulfadoxine–pyrimethamine for malaria in pregnancy: a facility-based, open-label, non-inferiority trial in Nigeria
Source: Malar J. 2018 Jul 6;17:251. doi: 10.1186/s12936-018-2394-2 (PMC6034215; doi:10.1186/s12936-018-2394-2)
Supplement: Supplementary file 4 — Additional file 4. Factors associated with low birth weight of babies delivered by study women (mITT analyses). [file 12936_2018_2394_MOESM4_ESM.docx]

**Additional file 4: Factors associated with low birth weight of babies delivered by study women (mITT analyses)**

|  | **Unadjusted RR** | **(95%CI)** | **p-value^a^** | **Adjusted RR^b^** | **(95%CI)** | **p-value** |
| --- | --- | --- | --- | --- | --- | --- |
| **Treatment group**  **ISTp-AL**  **IPTp-SP** | 0.95  1 | 0.40 to 2.21 | **0.898** | 0.94  1 | 0.40 to 2.25 | **0.896** |
| **Age category**  **≤ 25**  **26-30**  **≥ 31** | 1  1.16  0.88 | 0.41 to 3.28  0.26 to 2.92 | **0.781**  **0.828** | 1  1.41  1.53 | 0.49 to 4.05  0.43 to 5.40 | **0.52**  **0.507** |
| **Baseline parasitaemia**  **Yes**  **No** | 1.95  1 | 0.61 to 6.23 | **0.261** | 1.90  1 | 0.57 to 6.37 | **0.299** |
| **Baseline mild anaemia**  **Yes**  **No** | 1.25  1 | 0.53 to 2.97 | **0.612** | 1.19  1 | 0.49 to 2.93 | **0.7** |
| **Gravidity**  **Primigravidae**  **Secundigravidae**  **Multigravidae** | 3.44  2.13  1 | 0.80 to 14.89  0.42 to 10.67 | **0.098**  **0.359** | 3.96  2.13  1 | 0.84 to 18.61  0.41 to 11.12 | **0.082**  **0.371** |
